# Estimation of Measurement Uncertainties for the DGT Passive Sampler Used for Determination of Copper in Water

**DOI:** 10.1155/2014/389125

**Published:** 2014-09-01

**Authors:** Jesper Knutsson, Sebastien Rauch, Gregory M. Morrison

**Affiliations:** Department of Water Environment Technology, Division of Civil and Environmental Engineering, Chalmers University of Technology, 412 96 Göteborg, Sweden

## Abstract

Diffusion-based passive samplers are increasingly used for water quality monitoring. While the overall method robustness and reproducibility for passive samplers in water are widely reported, there has been a lack of a detailed description of uncertainty sources. In this paper an uncertainty budget for the determination of fully labile Cu in water using a DGT passive sampler is presented. Uncertainty from the estimation of effective cross-sectional diffusion area and the instrumental determination of accumulated mass of analyte are the most significant sources of uncertainty, while uncertainties from contamination and the estimation of diffusion coefficient are negligible. The results presented highlight issues with passive samplers which are important to address if overall method uncertainty is to be reduced and effective strategies to reduce overall method uncertainty are presented.

## 1. Introduction

The overall goal of environmental management programs is to provide a framework for assessing environmental status, identifying problem areas, and to continuously assess quality indicators to ensure that those are within established acceptable limits which ensure a “good and nondeteriorating status.” One of the indicators of environmental quality outlined by the Water Framework Directive of the European Union is heavy metal concentration in water bodies, including Cu, Pb, Cd, and Ni [1]. There is therefore a stated need to measure and assess the environmental concentration of these metals. This should be done using a method that is representative and that provides comparable results across EU member states, though the directive does not specify what level of uncertainty is considered sufficient.

A passive sampler is a device used to collect a target analyte* in situ*, both in gaseous and liquid media. Recently, passive samplers have found increasing use in the determination of metals and organic contaminants in water [2–4]. However, measurement uncertainty, relatively little investigated, is a perceived limitation of passive sampling in comparison to the more conventional grab and automated bottle sampling procedures. The work presented here aims at characterizing and assessing the uncertainty associated with the determination of time-weighted concentrations of labile metal ions in freshwater using passive sampling.

**Figure 1:**
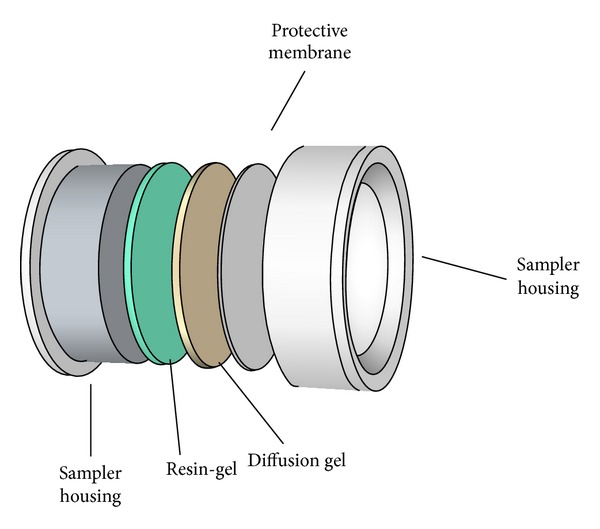
A schematic rendering of a DGT passive sampler showing its principal components.

A passive sampler for metal sampling is typically composed of a membrane filter, a diffusion layer gel, and a receiving phase placed in a sampler housing, like the DGT (diffusive gradients in thin films) technique (Figure 1). The DGT passive sampler was first described by Allan et al. [4] and since then the technique has been used in a wide range of applications and is one the most widely used passive sampler techniques for quantification and speciation of metals in aquatic environments. The analyte accumulates on the receiving phase as a result of the chemical affinity of the analyte for the solid receiving phase. The amount of analyte accumulated is proportional to the average concentration of labile analyte in the water, the time the sampler is exposed, and other aquatic environmental factors such as temperature and turbulence. After sampler retrieval and determination of the collected amount of metal, the average bulk concentration of metal can be calculated (see [3–5])

(1) $$\begin{array}{c} c_{b}=\frac{\left(M_{\text{acc}}-M_{\text{blank}}\right)\left(D^{w}\Delta g+D^{\text{MDL}}\delta \right)}{tD^{w}D^{\text{MDL}}A_{e}}. \end{array}$$

See [6].

In (1), *c*_*b*_ denotes the bulk concentration of the analyte in the water body, *M*_acc_ is the mass of the analyte accumulated on the sampler, *D* ^*W*^ is the diffusion coefficient of the analyte in water at 20°C, Δ*g* is the thickness of material diffusion layer (MDL, consisting of membrane filter and diffusion layer gel), *D* ^MDL^ is the diffusion coefficient of the analyte in the MDL, *δ* is the effective thickness of the diffusion boundary layer that is formed at the water-sampler interface, *t* is the time of exposure, and *A*_*e*_ is the effective sectional area of diffusion.

Although there has been some consideration of overall uncertainty in passive sampler measurements [7], there is no published study evaluating the components of this uncertainty. The identification of key components contributing to overall uncertainty can support the improvement of procedures based on passive samplings, as well as reducing potential concerns about performance and reliability [7].

## 2. Materials and Methods

For the purpose of this study, a simple case was assumed; a DGT passive sampler with characteristics listed in Table 1 was used to determine dissolved Cu concentration in water (*c*_*b*_ = 1.28 ± 0.16 × 10^−6^ gl^−1^). We note that the estimation of uncertainty resulting from metal-ligand interactions is out of the scope of this paper and Cu is therefore considered fully labile and present as Cu^2+^. In the absence of metal complexes, the time weighted average concentration can be derived using (1).

The uncertainty budget presented here was estimated for a generic passive sampler under predefined environmental conditions (Table 1). The characteristics of the passive samplers were chosen based on the characteristics of existing commercially available samplers (DGT Research Ltd.) and the availability of data. Similarly, environmental conditions were selected based on the availability of data for specific samplers. Although a number of passive sampler technologies have been described in the literature [8–11], the general methodology presented in this work should be applicable to estimate measurement uncertainty for a broad range of passive samplers, even if the specific conclusions for the passive sampler system assessed here does not necessarily hold true for other types.

**Figure 2:**
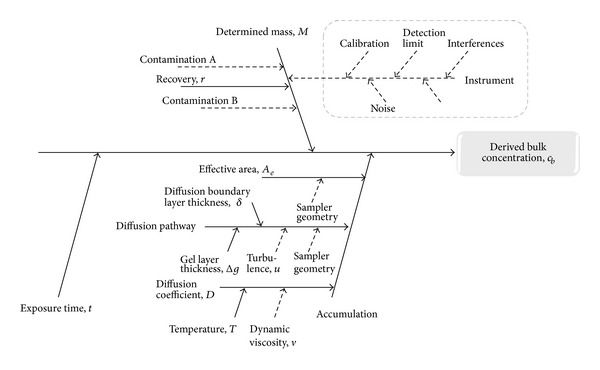
Cause and effect diagram describing the uncertainties associated with the determination of bulk concentration *c*_*b*_, using a passive sampler. Dashed arrows indicate parameters whose uncertainty contribution was included in another parameter. The dashed box shows the uncertainty from instrument determination of analyte. Uncertainty analysis of the ICP-MS technique has been performed previously [19, 22] and was therefore not treated separately in this paper.

Uncertainty in passive sampling is expected from all steps in the analytical process, including preparation of the samplers, deployment, analyte extraction, analysis, and estimation of diffusion rates and pathways. Overall, the estimation of uncertainties and the propagation of uncertainties were based on standard methodology [12]. Input data for the calculation were obtained from the literature and our own results, depending on availability. A cause and effect diagram was created to visualize the sources of uncertainty in the analytical chain when using a passive sampler to determine time weighted average bulk concentration (Figure 2). A list of relevant parameters (see Table 2) was identified from the cause and effect diagram and the model equation as a basis for the construction of the uncertainty budget.

## 3. Results and Discussion

### 3.1. Uncertainties in Analyte Accumulation

#### 3.1.1. Diffusional Pathway

**Figure 3:**
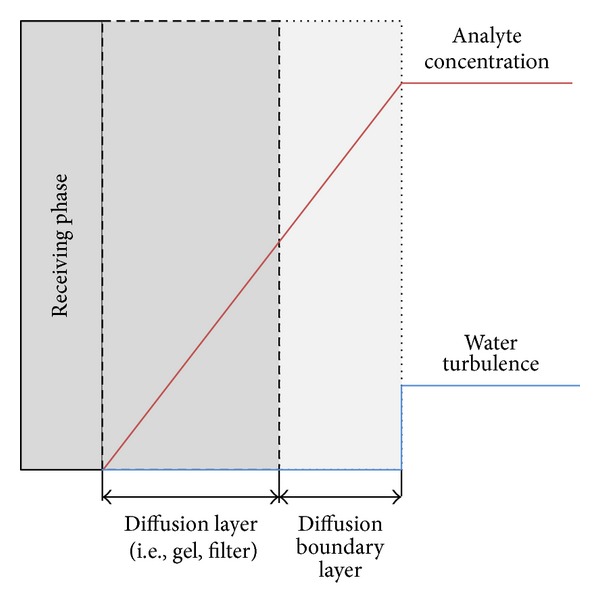
Schematic representation of the concentration gradient that forms over the diffusional pathway.

When deploying a prepared passive sampler, the fully labile metal ion (Cu^2+^) accumulates on the receiving phase and the accumulation rate is governed by diffusion across a diffusion boundary layer (DBL, see Figure 3), a membrane filter of known thickness (0.135 mm), and a gel layer of a known thickness (0.80 mm). No assessment was found of the uncertainty of Δ*g*, but a low uncertainty level was assumed for the combined membrane filter and gel layer (0.935 ± 0.05 mm) based on the authors judgement [12].

The DBL is the water layer closest to the passive sampler-water interface that is not affected by the mixing conditions in the bulk water phase. This measure is a representation of the effective DBL as this is neither evenly distributed layer across the surface nor a true unmixed layer but rather a velocity gradient. The effective thickness of the DBL is subject to uncertainty. The uncertainty can be reduced by deploying several devices with varying Δ*g*, as described by Zhang et al. [13], but this procedure increases the scope and cost of measurement considerably. Therefore in the hypothetical scenario presented here, the DBL thickness was estimated to be 0.26 ± 0.05 mm, covering a wide range of flow regimes, from fast flowing water to slow moving lake epilimnion [6]. The diffusion coefficient of the metal ion Me^2+^ depends in turn on the water temperature and on which media it is diffusing in. The total accumulated amount (*M*) depends on the accumulation rate and the length of the exposure in time (*t*).

#### 3.1.2. Diffusion Coefficients

The diffusion coefficients *D* ^*W*^ and *D* ^MDL^ are usually determined experimentally in a separate experiment. The determination itself is associated with uncertainty, and results are typically reported without associated uncertainty. A typical relative uncertainty of diffusion coefficients has been reported in the range 1.3–6.4% [14, 15] and for the purpose of this assessment we will use a *D* ^MDL^ value of 6.42 × 10^−10 ^m^2^ s^−1^, which is the diffusion coefficient of Cu^2+^ in APA2 gel (a polyacrylamide hydrogel containing 15 % vol acrylamide and 0.3% agarose-derived cross-linker) and the upper value in the uncertainty interval, that is, 6.4% [15]. The diffusion coefficient of Cu^2+^ in water (*D* ^*W*^) is reported to be 1.14 times larger at 7.30 × 10^−10 ^m^2^ s^−1^ [15]. For the purpose of this paper that same relative uncertainty was applied to both *D* ^MDL^ and *D* ^*W*^. It should be noted that effective diffusion coefficients may also be significantly affected in low ionic strength solutions (<1 mM).

The diffusion coefficient *D* depends on water temperature as described by the Stokes-Einstein equation:

(2) $$\begin{array}{c} D=\frac{k_{b}T}{3\pi \mu d}, \end{array}$$

where *k*_*b*_ is the Boltzmann constant (m^2^ kg s^−2^ K^−1^), *T* is the temperature (K), *μ* is the viscosity of the medium (kg s^−1 ^m^−1^), and *d* is the spherical diameter of the diffusing particle.

The uncertainty introduced from variability of *T* was analysed (*D*(*T*)). The uncertainty in the experimental determination of *D* was also estimated. The standard uncertainty in *D* ^*W*^ from uncertainty in water temperature was calculated to be 0.06 × 10^−10^ m^2^ s^−1^, and the combined standard uncertainty from the determination of *D* ^*W*^ and temperature was calculated through summation in quadrature to be 7.30 ± 0.47 × 10^−10^ m^2^ s^−1^. A similar treatment of *D* ^MDL^ resulted in 6.42 ± 0.10 × 10^−10^ m^2^ s^−1^.

#### 3.1.3. Effective Area

The effective area of the section through which diffusion occurs has been reported to be somehow larger than the nominal area due to lateral diffusion; that is, diffusion occurs in three dimensions [6, 7]. Warnken et al. report that the radius of the effective diffusion window is 1.02 ± 0.024 cm and also note that the gel disc diameter had shrunk on average 0.12 cm (*n* = 6) during drying prior to determination of the radius [16]. No estimate on uncertainty was given for this measure, so a 0.05 cm uncertainty was assumed based on the number of significant figures reported, and a rectangular distribution was selected due to the lack of information on the measurement.

Summation in quadrature was used to combine the uncertainties from the determination of effective radius and the estimation of the shrinkage in order to calculate the total uncertainty associated with the effective area [17]. The divisor $\sqrt{3}$ was used to get the standard uncertainty of the shrinkage because of the assumed rectangular distribution, followed by summation in quadrature:

(3) $$\begin{array}{c} U_{c}=\sqrt{u_{i}{\left(r_{\text{disc}}\right)}^{2}+{\left(\frac{u_{i}\left(r_{\text{shrinkage}}\right)}{\sqrt{3}}\right)}^{2}}. \end{array}$$

The combined uncertainty of the effective radius was calculated to be 0.0449 cm, making the effective radius of the sampler 1.08 ± 0.04 cm. Using the derivative of the circle area function to calculate the uncertainty of the effective area, *A*_*e*_, gave the value 3.66 ± 0.30 cm^2^.

### 3.2. Uncertainties in Determination of Mass

#### 3.2.1. Preparation and Handling

During preparation, transport, storage, and handling of the passive sampler devices there is a risk of contamination. The best assessment of the uncertainty from these sources comes from the use of field blanks [18]. The field blanks can be used to correct for contamination issues. We have estimated during field trials that the associated relative uncertainty resulting from contamination is typically in the order of 24% for passive sampler devices, with field blank values of 8.1 ± 2.0 ng Cu^2+^ (*n* = 3) (unpublished data).

#### 3.2.2. Extraction

The analyte (Cu^2+^) is subsequently extracted from the receiving phase using a small volume of nitric acid. The recovery factor, *r*, has been reported previously (0.793 ± 0.051) [5]. The uncertainty was reported as an interval, and therefore a rectangular distribution was assumed.

#### 3.2.3. Analysis/Determination

The resulting extract is diluted to a suitable volume concentration before analysis by a selected analytical technique. Inductively coupled plasma-mass spectrometry (ICP-MS) is widely used for the determination of trace metal concentrations in environmental samples and therefore, we estimate uncertainty for ICP-MS analysis in this paper. The ICP-MS instrument is calibrated using calibration standards prepared from certified standard solutions.

Generally, the analytical procedure using ICP-MS is subject to known and unknown interferences of which some can be compensated for, while others may persist, depending on specific instrument capabilities [18]. Furthermore, instrument drift, stability of stock solutions, and density of stock solutions will contribute to uncertainty [18] and the uncertainty budget of the instrumental analysis is a comprehensive topic in its own right. A simplified view is given in Figure 2 to highlight the importance of the analytical step. However, instrument performance and the typical uncertainty of the method have been addressed elsewhere [19] and are not repeated here. The reported standard relative uncertainty for Ni solutions containing 10 ng g^−1^ or more was 7.5%, which was used for the calculations in this paper.

The estimated accumulated mass and mass on blank samples was determined using ICP-MS and then corrected for by the recovery factor according to

(4) $$\begin{array}{c} M_{\text{acc}/\text{blank}}=\frac{m_{\text{icp-ms}}}{r}. \end{array}$$

Using the rule for uncertainty propagation in quotients the estimate for *M*_acc_ becomes 1.63 ± 0.06 × 10^−6^ g (see Table 3). A similar treatment of *M*_blank_ resulted in 0.010 ± 0.003 × 10^−6^ g.

### 3.3. Total Combined Uncertainty of the Passive Sampler Measurement

To estimate the combined standard uncertainty of the bulk concentration *c*_*b*_, the relation in the model equation (1) was used. Since it was a mixed expression, the rule of uncertainty propagation states that the combined uncertainty can be calculated using

(5) $$\begin{array}{c} \partial Q=\sqrt{{\left(\frac{\partial q}{\partial x}\delta x\right)}^{2}+\cdots +{\left(\frac{\partial q}{\partial z}\delta z\right)}^{2}}. \end{array}$$

See [20].

This means that the combined uncertainty is equal to the root square sum of the partial derivatives of the variables. However, it is also possible to derive a numerical solution as suggested by Kragten [21]. The approximation derived from this numerical method assumes linearity and small values of relative uncertainty, *u*(*x*_*i*_)/*x*_*i*_. While this is not always the case, the accuracy of the solution is still acceptable for most practical purposes [21].

A summary of the quantities and the associated standard uncertainties is presented in Table 4.

During calculations values were not rounded to avoid the introduction of additional uncertainty. The output of the numerical treatment of combined uncertainties can be seen in Table 5. The measurement output with associated uncertainty was *c*_*b*_ = 1.32 ± 0.100 *μ*g l^−1^. Using a coverage factor *k* = 2 the result was instead 1.32 ± 0.200 *μ*g l^−1^ (confidence interval ≈ 95%), or a relative uncertainty of 7.6% at *k* = 1.

**Figure 4:**
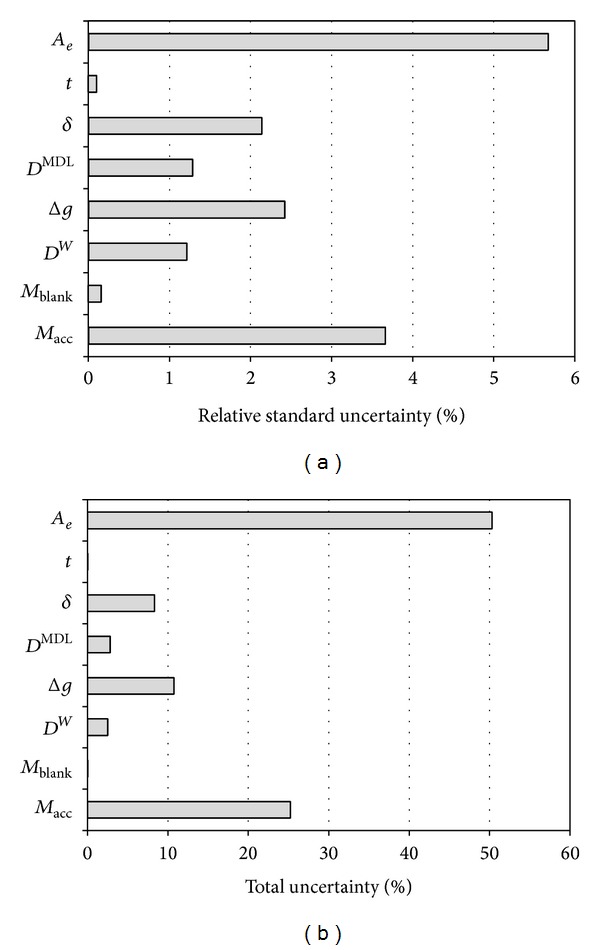
Relative standard uncertainty (a) and percentage of total uncertainty (b) for the variables in the model equation.

When plotting the relative standard uncertainties of the components graphically (Figure 4) it is obvious that the largest uncertainty was introduced from the effective cross-sectional area estimate (*A*_*e*_). The combined estimated uncertainties resulting from the determination of the lateral diffusion round the edges and the shrinkage of the gel resulted in an uncertainty that largely affects the end result, as it accounts for nearly 50% of the total uncertainty (see Figure 4). Uncertainties from the estimation of *M*_acc_ account for roughly 25% of the total uncertainty, with the most significant factor being the estimation of extraction recovery.

A sensitivity analysis shows that halving the uncertainty for the effective radius and shrinkage in the determination of *A*_*e*_ would reduce the contribution of *A*_*e*_ to the overall uncertainty to roughly 20% (1.32 ± 0.08 *μ*g l^−1^ or 6.0% relative overall uncertainty). Similarly, a reduction in the uncertainty in the recovery factor, *r*, by 50%, would reduce the contribution from *M*_acc_ to overall uncertainty from 25% to approximately 9% (1.32 ± 0.09 *μ*g l^−1^ or 6.8% relative overall uncertainty). On the other hand, an increase in the uncertainty for diffusion layer thickness from 0.05 mm to 0.2 mm would result in 1.32 ± 0.16 *μ*g l^−1^ or 12.2% relative overall uncertainty. This is a significant increase in overall uncertainty and illustrates the sensitivity of the method to inconsistencies in the gel-membrane layer interface. Furthermore, the effects of uncertainty changes in DBL and diffusive layer thickness are shown in Table 6.

The sensitivity analysis shows that overall method uncertainty can be significantly reduced by addressing the proper sources of uncertainties and also that deterioration in diffusion layer consistency can have significant negative effects on overall method uncertainty.

## 4. Conclusion

An uncertainty analysis was performed for passive sampling of a metal ion in water to highlight critical steps in the method and to identify key factors for potential improvement. In the analysis performed here the uncertainty of the effective cross-sectional diffusion area *A*_*e*_ was identified as the main contributor to overall uncertainty. Uncertainties in analyte recovery and material diffusion ranked second and third, respectively. An improvement in the estimation of *A*_*e*_ was found to be an important step toward achieving a reduction in uncertainty in passive sampling. Optimization of the extraction procedure will provide a further reduction in overall uncertainty.

## Figures and Tables

**Table 1:** Predefined passive sampler characteristics and environmental conditions used as a basis in the uncertainty calculations.

Parameter	Property/Value
Passive sampler	
Diameter	2 cm
Diffusion layer	Acrylamide gel with APA cross-linker (APA2) [23]
Cellulose nitrate membrane	135 *µ*m thickness and 0.45 *µ*m pore size
Receiving phase	Resin-gel containing Chelex resin
Environmental conditions	
pH	7.5
Water temperature	25°C/298 K
Turbulence	Estimated

**Table 2:** Parameters for which uncertainty is determined and respective units.

Parameter	Unit	Definition
*A*_*e*_	m^2^	Effective area of diffusional cross-section
*D* ^MDL^	m^2^ s^−1^	Diffusion coefficient of the Cu^2+^ ion in the MDL
*D* ^*W*^	m^2^ s^−1^	Diffusion coefficient of the Cu^2+^ ion in water
*M*	g	Accumulated amount of Cu^2+^ determined from sample
*M*_blank_	g	Contamination determined from field blank
*r*		Recovery during the extraction phase
*T*	K	Temperature in bulk water phase
*t*	hours	Exposure time
*δ*	m	Diffusional boundary layer thickness
Δ*g*	m	Diffusional pathway thickness of the MDL

**Table 3:** Uncertainty budget for *M*_acc_ showing relative uncertainties for the variables and the combined standard uncertainty.

Symbol	Source of uncertainty	Type∗	Standard uncertainty *u*(*x*_*i*_)	Distribution	Divisor	Relative uncertainty
*m*_icp-ms_	Estimated mass from ICP-MS analysis	A	1.0 × 10^−8^ g	Normal	1	0.008
*r*	Recovery factor	B	0.0293	Rectangular	$\sqrt{3}$	0.064
Uc (M)	Combined standard uncertainty	A	6.15 × 10^−8^ g	Normal		0.038

*Note: type of uncertainty refers to types A and B, using standard vocabulary for statistically evaluated uncertainty (A) and uncertainty evaluated by other methods (B).

**Table 4:** Quantities, nominal values, and their associated uncertainty used in this work.

Quantity	Value	Standard uncertainty	Comment
*A*	3.66 cm^2^	0.30 cm^2^	See previous section and [16]
*D* ^MDL^	6.42 × 10^−10^ m^2^/s	0.09 × 10^−10^ m^2^/s	Empirical value [15]
*D* ^*W*^	7.30 × 10^−10^ m^2^/s	0.47 × 10^−10^ m^2^/s	Empirical value [15]
*M*_acc_	1.29 × 10^−6^ g	0.01 × 10^−6^ g	Observation
*M*_blank_	0.008 × 10^−6^ g	0.002 × 10^−6^ g	Observation
*r*	0.793	0.051	Observation [5]
*t*	168 h	0.3 h	Covers the time it takes to deploy and retrieves 5 passive samplers
*T*	25°C/298 K	4 K	Standard deviation of the measured temperature
*δ*	0.26 × 10^−3^ m	0.05 × 10^−3^ m	Estimate [16]
Δ*g*	0.9 × 10^−3^ m	0.05 × 10^−3^ m	Estimate

**Table 5:** Uncertainty budget for determination of time weighted average concentration of Cu^2+^ in water using a DGT passive sampler.

Symbol	Source of uncertainty	Type	Standard uncertainty *u*(*x*_*i*_)	Distribution	Divisor	*U*_*i*_ (M) *μ*g L^−1^
*M*_acc_	Determination of accumulated mass	A	6.14 × 10^−8^ g	Normal	1	0.49
*M*_blank_	Determination of contamination	A	2.55 × 10^−9^ g	Normal	1	0.02
*D* ^*W*^	Diffusion coefficient in water	A	4.73 × 10^−11^ m^2^/s	Normal	1	0.16
Δ*g*	Thickness of material diffusion layer (MDL)	B	2.89 × 10^−5^ m	Rectangular	$\sqrt{3}$	0.33
*D* ^MDL^	Diffusion coefficient in MDL	A	1.03 × 10^−11^ m^2^/s	Normal	1	0.16
*δ*	Diffusion boundary layer	B	2.89 × 10^−5^ m	Rectangular	$\sqrt{3}$	0.29
*t*	Time	B	624 s	Rectangular	$\sqrt{3}$	0.01
*A*_*e*_	Effective area	A	2.08 × 10^−5^ m^2^	normal	1	0.69
Uc (*c*_*b*_)	Combined standard uncertainty			Normal		0.98
Uc (*c*_*b*_)	Expanded standard uncertainty			Normal (*k* = 2)		1.95

**Table 6:** Results from sensitivity analysis, showing the effect on total uncertainty of the passive sampler measurement from reductions in uncertainty of selected parameters.

Parameter	Change in uncertainty	Result on total uncertainty
Effective area, *A*_*e*_	50% reduction	Reduction from 7.6% to 6.1% in overall relative uncertainty
Recovery factor, *r*	50% reduction	Reduction from 7.6% to 6.9% in overall relative uncertainty
Diffusion boundary layer, *δ*	From 0.05 mm to 0.014 mm standard uncertainty	Reduction from 7.6% to 7.3% in overall relative uncertainty
Diffusion pathway thickness	50% reduction	Reduction from 7.6% to 7.3% in overall relative uncertainty
Diffusion pathway thickness	4 times increase	Increase from 7.6% to 12.2% in overall relative uncertainty
